# Enhancing evidence-based care using trial emulation in electronic health records: real-world effects of empagliflozin in people with type 2 diabetes

**DOI:** 10.1136/bmjdrc-2025-005672

**Published:** 2026-02-10

**Authors:** David K Ryan, Ruth H Keogh, Elizabeth Williamson, R Thomas Lumbers, Karla Diaz-Ordaz, Anoop D Shah, Patrick Bidulka

**Affiliations:** 1 Institute of Health Informatics, University College London, London, UK; 2 Medical Statistics, London School of Hygiene and Tropical Medicine, London, UK; 3 Institute of Health Informatics, UCL, London, UK; 4 Department of Statistical Science, University College London, London, England, UK; 5 Non-Communicable Disease Epidemiology, London School of Hygiene and Tropical Medicine, London, UK

**Keywords:** Cardiology, Epidemiology

## Abstract

**Background:**

There is growing interest in widening the use of sodium-glucose co-transporter 2 inhibitors (SGLT2i) to all people with type 2 diabetes mellitus (T2DM). However, pivotal randomized controlled trials (RCTs) evaluated these drugs only in highly selected populations, often lacking generalizability to real-world populations. Understanding the effects of SGLT2i in populations where RCT evidence may be lacking is essential to help inform guideline development. To address this, we estimated the effect of empagliflozin in real-world users, many of whom would not have been eligible for the pivotal EMPA-REG RCT.

**Methods:**

We designed a trial emulation in UK primary care data, based on the EMPA-REG RCT, to assess the effect of empagliflozin in a more clinically relevant population. Adults with T2DM initiating empagliflozin (intervention) or dipeptidyl peptidase-4 inhibitors (active control) between January 1, 2014 and December 31, 2022 were included. Eligibility was extended to both RCT-eligible and RCT-ineligible individuals. The effect of empagliflozin on all-cause mortality was estimated using an adjusted Cox proportional hazards model, with stratified analyses by RCT eligibility.

**Findings:**

The majority of people prescribed empagliflozin would not have met the EMPA-REG RCT eligibility criteria (11,011/13,239, 83.2% RCT-ineligible). During follow-up, all-cause mortality occurred in 551 out of 13,239 (4.2%) in the empagliflozin group and 6,589 out of 49,264 (13.4%) in the active control group (adjusted HR 0.76, 95% CI 0.69 to 0.83). There was no evidence of differential treatment effect by RCT eligibility status (p-interaction=0.27).

**Interpretation:**

Patients prescribed empagliflozin in real-world settings differ substantially from those enrolled in the EMPA-REG RCT. Using electronic health records, we demonstrate that the mortality benefit observed in EMPA-REG extends to a broader, more diverse real-world population, including those excluded from the original RCT. These findings provide a novel source of real-world evidence supporting the wider use of empagliflozin in routine clinical practice.

**WHAT IS ALREADY KNOWN ON THIS TOPIC**

- Randomized controlled trials (RCTs), such as the EMPA-REG RCT, have shown that empagliflozin significantly reduces mortality in people with type 2 diabetes (T2DM) and established atherosclerotic cardiovascular disease, compared with standard care.
- UK and international guidelines now recommend broader use of empagliflozin for T2DM management beyond the populations originally enrolled in RCTs.
- Due to the divergence between RCTs and clinical practice, the real-world effectiveness of empagliflozin in current users is unclear.

**WHAT THIS STUDY ADDS**

- Using the trial emulation framework, we demonstrate that relaxing the stringent RCT eligibility criteria imposed by EMPA-REG RCT did not alter the treatment effect in real-world populations.
- This study provides a novel evidence base for many people living with T2DM, who are not represented in clinical trials.

**HOW THIS STUDY MIGHT AFFECT RESEARCH, PRACTICE OR POLICY**

- These results support broader use of empagliflozin in T2DM management and highlight the role of electronic health records in expanding and enhancing evidence-based care.

## Introduction

 Type 2 diabetes mellitus (T2DM) is a growing global health concern. In the UK, over 3.5 million people live with T2DM, and by 2040, the prevalence of T2DM is estimated to increase by 50%.^1^ Since 2022, the UK National Institute for Health and Care Excellence (NICE) treatment guidelines for T2DM recommend metformin and sodium-glucose co-transporter 2 inhibitors (SGLT2i) as dual first-line agents for a large number of people with T2DM.^2^ This includes people with T2DM and concomitant cardiovascular disease (CVD), risk factors for CVD, or heart failure. The 2022 NICE guideline committee decision was based on extrapolations from randomized controlled trials (RCTs), such as the EMPA-REG RCT, which showed significant cardioprotective and mortality benefits of SGLT2i.^2^ This follows similar recommendations by the European Society of Cardiology,^3^ European Association for the Study of Diabetes, and the American Diabetes Association.^4^

EMPA-REG RCT was a large multicenter trial that randomized 7,020 people with T2DM and established CVD to receive either the SGLT2i, empagliflozin, or placebo.^5^ To be eligible for this study, people had to meet stringent eligibility requirements. For example, participants were required to have evidence of established CVD such as a previous myocardial infarction or unstable angina with evidence of coronary artery disease on coronary angiogram. This focus on participants with high CVD burden was consistent across other major SGLT2i trials.^6^,^8^ However, NICE and other bodies subsequently opted to recommend SGLT2i for people with T2DM who did not have established CVD, such as a previous cardiac event, but were considered at high risk for CVD.^2^,^4^ This lowered the threshold of CVD required for treatment and expanded the treatment-eligible population compared with the RCT.^9^ Due to the differential use of this drug in current clinical practice compared with the RCTs, the real-world benefits of SGLT2i in T2DM management remain uncertain.

There is now a growing interest in using observational data to generate real-world evidence for treatments, either to complement existing research or fill gaps in the current knowledge base.^10^,^12^ In addition, real-world evidence can give insights that may not be studied, or indeed infeasible to study, through RCTs alone, such as long-term effects of medications, treatment effects in trial-excluded populations, heterogeneous treatment effects, as well as comparative effectiveness and safety studies, among others.^10^,^12^

Using a trial emulation framework, we leverage UK primary care data to estimate the real-world treatment effect of a key anti-diabetes drug in a broader population compared with those enrolled in RCTs. The aim of this study is to explore whether the mortality benefit observed in EMPA-REG extends to a broader, more diverse real-world population, including those excluded from the original RCT. In our emulation, we modified the design of the EMPA-REG RCT to study the real-world treatment effect of empagliflozin in broader populations which better reflect current clinical practice. We subsequently estimate the treatment effect of empagliflozin among initiators who would have been excluded for the EMPA-REG RCT, thereby generating evidence for empagliflozin use among the large population currently under-represented in pivotal RCTs.

## Methods

### Data sources

We used deidentified UK primary care electronic health record data from The Health Improvement Network (THIN), a Cegedim database.^13 14^ THIN contains data relating to demographics, lifestyle factors, diagnoses, prescribed medication, examination findings, laboratory, and other clinical measurements (blood pressure, body mass index, lipid profiles), and is representative of the UK population. Within the THIN database, diagnoses are recorded using Read codes, which are a standardized clinical terminology system. This study meets all of the CODE-EHR standards for the use of structured healthcare data in clinical research (online supplemental material 13).^15^

### Study design

We designed a trial emulation which adapts the design of the EMPA-REG RCT. Specifically, we relaxed the stringent eligibility criteria imposed by the RCT to capture the broader population of real-world empagliflozin initiators and better reflect current clinical practice. This study was designed with reference to guidelines for best practice in trial emulation^16^ and is described in table 1. It is not possible to directly emulate a placebo-controlled trial; therefore, we employed a new-user active comparator design to compare mortality between people initiated on empagliflozin versus people initiated on dipeptidyl peptidase-4 inhibitors (DPP4i, which were defined as: alogliptin, linagliptin, sitagliptin, saxagliptin, and vildagliptin). An active comparator was selected to help mitigate confounding by indication. DPP-4i drugs were selected as the active comparator as they have no known cardioprotective or mortality benefit^17^ and were a common therapeutic alternative to SGLT2i during the study period.

**Table 1:** Study design of the RCT and trial emulation

	EMPA-REG RCT	Trial emulation
Inclusion criteria	Individuals with T2DM, aged over 18 years, with established coronary artery disease (previous acute coronary syndrome, established coronary artery disease), stable glycemic control and BMI ≤45 kg/m^2^	Individuals with T2DM, aged over 18 years, who are SGLT2i and DPP-4i naïve and have an incident prescription for either empagliflozin or DPP-4i between January 1, 2014 and December 31, 2022
Exclusion criteria	People with baseline renal impairment (eGFR <30 mL/min/1.73 m^2^), recent acute coronary syndrome, stroke or transient ischemic attack, liver disease, recent pregnancy among other factors. See supplementary material for full list	Medical history of pancreatitis or ketoacidosis
Treatment strategies	Empagliflozin or placebo	Incident initiators of either empagliflozin or DPP-4i (active comparator design)
Treatment assignment	Randomization with analysis via intention-to-treat	Analyzed as observational analog of intention-to-treat
Day zero	Date of randomization following a 2-week open-label placebo run-in period	Date of first incident prescription for either empagliflozin or DPP-4i between January 1, 2014 and December 31, 2022, whichever occurred first
Follow-up period	Median follow-up of 3.1 years	Follow-up begins at the day zero and ends at first of: date of death, date of leaving general practice or December 31, 2023
Confounders	Adjusted for chance baseline differences between groups (age, gender, baseline BMI, baseline HbA1c, baseline eGFR, geographical region)	Adjusted for confounding variables at baseline in an adjusted Cox proportional hazards model
Outcomes	Primary outcome: time-to-first composite outcome event (cardiovascular death, non-fatal stroke, non-fatal myocardial infarction) Secondary outcome: time-to all-cause mortality	Primary outcome: time-to all-cause mortality
Causal treatment effect	Adjusted HRs for composite outcome and all-cause mortality, both estimated by a Cox proportional hazard models	Adjusted HR for all-cause mortality estimated via a Cox proportional hazards model

The table highlights the main design elements of the trial emulation, with corresponding design of the EMPA-REG RCT.

BMI, body mass index; DPP-4i, dipeptidyl peptidase-4 inhibitors (alogliptin, linagliptin, sitagliptin, saxagliptin and vildagliptin); eGFR, estimated glomerular filtration rate; HbA1c, glycated hemoglobin A1c; RCT, randomized controlled trial; SGLT2i, sodium glucose co-transporter 2 inhibitor (empagliflozin, dapagliflozin, canagliflozin and ertugliflozin); T2DM, type 2 diabetes mellitus.

Trial emulation is a methodological approach that replicates either an existing or a hypothetical clinical trial in an observational setting.^16 18^ The purpose of the trial emulation framework is to reduce bias in observational studies by pre-specifying eligibility criteria, time zero, treatment strategies, follow-up and outcome measures and is advocated by the UK NICE.^11^ A key benefit of targeting an existing RCT is that the observational results can be ‘benchmarked’ to the published results of the RCT, giving confidence that the real-world analysis is accounting for confounding appropriately. This can then support the emulation of hypothetical or modified trials, allowing us to draw meaningful inferences about causal effects of medications in real-world practice.

The present trial emulation is primarily designed as a hypothetical trial to study treatment effects in people who are now receiving empagliflozin in routine clinical practice. It is informed by the EMPA-REG RCT, but studies a much broader, more clinically relevant population of people with T2DM. We also compare the treatment effect in RCT-eligible and RCT-ineligible initiators of empagliflozin, thereby enabling the results to be benchmarked against the published results of the EMPA-REG RCT.

### Study population

The trial emulation analyzed data from adults with T2DM who had an incident prescription for either empagliflozin or an active control, DPP-4i between January 1, 2014 and December 31, 2022. These study dates were chosen to align with the introduction of SGLT2i in the UK and the subsequent updates to NICE guidelines in June 2022, which thereafter prioritized SGLT2i as an oral anti-diabetic agent.^19^ People who had prior use of a SGLT2i or DPP-4i medication before cohort entry date were excluded to avoid dilution of treatment effect from a drug-class effect. People were analyzed according to their treatment assignment on day zero in an intention-to-treat manner, regardless of post-baseline changes in treatment assignment (eg, discontinuation, switching, or intensification).

Unlike the stringent eligibility criteria defined in the EMPA-REG RCT,^5^ we did not exclude individuals based on criteria such as presence of established atherosclerotic disease, glycated hemoglobin A1c (HbA1c), BMI, or renal function thresholds. The only inclusion criteria we applied was being aged over 18 years and a confirmed diagnosis of T2DM.

People were excluded from the trial emulation if they registered with their general practice within one year of cohort entry to ensure sufficient capture of baseline covariates. People were also excluded if they had a history of pancreatitis or ketoacidosis because they would have a low chance of being prescribed an SGLT2i due to clinical recommendations advising caution in prescribing SGLT2i to such individuals.^20^

### Outcomes

The primary outcome in the EMPA-REG RCT was a composite three-point major adverse cardiovascular event, consisting of CVD death, non-fatal myocardial infarction, and non-fatal stroke.^5^ However, cause-specific mortality data were not available in the THIN database. As a result, the primary outcome for the trial emulation was all-cause mortality. This was reported as a secondary outcome in the EMPA-REG RCT, allowing for comparison of results. The date of death was estimated using a validated algorithm,^21^ which gathers various recorded death codes from primary care records and integrates the data to determine the most accurate estimated date of death. Follow-up begins at the date of first prescription of empagliflozin or DPP-4i and ends at first of: date of death, date of leaving general practice or December 31, 2023.

### Confounders

Confounders were identified based on clinical knowledge and included age, sex, ethnicity, socioeconomic status (defined by the index of multiple deprivation quintile at the level of a GP practice), calendar year of cohort entry, comorbidities, co-prescribed medication, and laboratory and clinical measurement values (blood pressure, BMI, HbA1c, cholesterol), among others. These are summarized in a directed acyclic graph (online supplemental material 1). Pre-existing published codelists from the Health Data Research UK phenotype library were used to define covariates. Details and codelists for covariates are described in the online supplemental material 2 and 3. Ethnicity was re-categorized according to the most recent UK census classification (online supplemental material 3).^22^

HbA1c was defined based on the most recent value up to 180 days before time zero. This 180-day window was selected as NICE recommends that HbA1c is measured every 6 months in people with T2DM.^19^ For other measurements such as systolic blood pressure, BMI, cholesterol, and estimated glomerular filtration rate (eGFR), the most recent value within a window of 540 days before baseline was used. This was informed by the UK primary care Quality and Outcomes Framework, which recommends patients with T2DM have a full clinical review annually, with additional time allocated for delays and data entry. This is consistent with approaches taken by others studying real-world drug effects for T2DM.^23^ If values were not recorded, or not available within the eligible window for the covariate in question, the value was defined as missing. Further details are available in online supplemental material 2. Other covariates were defined based on the most recently available information at day zero.

### RCT eligibility status for empagliflozin users

Once our trial emulation population was defined, we assessed what proportion of these people would have met the stringent eligibility criteria of the EMPA-REG RCT. The mapping of the RCT eligibility criteria to the observational setting is described in online supplemental material 5 table 3. This enabled us to define RCT eligibility status for each user of empagliflozin or DPP-4i, based on whether they would have met the EMPA-REG RCT criteria. Several RCT eligibility criteria related to variables that had missing values such as laboratory tests at baseline (eg, estimated glomerular filtration rate, baseline HbA1c). People were defined as RCT eligible if these values were missing or not measured within the predefined window before cohort entry date.

### Statistical analysis

Full details are in the online supplemental Appendix. Demographics, clinical measurements, prescribed medication, and comorbidities were described for the trial emulation population overall, and by treatment group, and were compared with the published table of subject characteristics from the EMPA-REG RCT.

An adjusted Cox proportional hazard model was used to estimate the HR for all-cause mortality, with time on study as the timescale. The Cox proportional hazards model was adjusted for confounders that are defined in the study directed acyclic graph. BMI, low-density lipoprotein (LDL) and high-density lipoprotein (HDL) cholesterol, eGFR, HbA1c, systolic blood pressure, ethnicity, and smoking status had missing data. Missing values were handled using multiple imputation by chained equations,^24 25^ generating five fully imputed datasets with pooled estimates derived using Rubin’s rules.

Three-year risk difference in all-cause mortality between treatment groups and associated number needed to treat (NNT) to prevent one death were estimated using a g-computation approach, with confidence intervals (CIs) estimated using a bootstrap imputation procedure.^26^ Three years of follow-up was selected as this matches the median follow-up period of the EMPA-REG RCT.^5^ For sensitivity analysis, an inverse-probability of treatment weighted (IPTW) Cox proportional hazard model was employed to estimate the average treatment effect and also the average treatment effect on the treated.

We then estimated the real-world treatment effect of empagliflozin, stratified according to RCT eligibility status, by adding an interaction term to the adjusted Cox proportional hazards model. This tested whether the treatment effect differed by RCT eligibility status and provided a HR for the effect of empagliflozin versus DPP-4i in both RCT-ineligible and RCT-eligible initiators of empagliflozin.

Results from the RCT eligible subgroup enabled benchmarking against EMPA-REG RCT estimates using predefined agreement metrics. These metrics are consistent with other trial emulations^27^:

1. Statistical significance agreement: Emulated estimates and CIs align on the same side of the null as the RCT.
2. Estimate agreement: Emulated estimates fall within the 95% CIs of the RCT estimate.
3. Standardized difference: Compares the difference in effect size between the RCT and trial emulation, allowing formal hypothesis testing.

### Sensitivity analysis

We assessed bias using E-values, which quantify the minimum strength of association a binary unmeasured confounder would need to have an association with both the treatment and the outcome to fully explain away the observed association^28^ and can give insight into whether the findings are robust to potential unmeasured confounding.

### Patient and public involvement

We presented the study at a patient and public workshop with nine participants, alongside a wider discussion of the use of electronic health records to determine real-world drug effects. Participants were supportive of the study and were surprised at the lack of representativeness in clinical trials.

## Results

### Trial emulation population and baseline characteristics

A total of 62,503 people with T2DM initiated either empagliflozin (n=13,239, 21.2%) or DPP-4i (n=49,264, 78.8%) between January 1, 2014 and December 31, 2022. A total of 7,140 deaths (11.4%) were recorded in this trial emulation. Death occurred in 551 out of 13,239 (4.2%) of the empagliflozin group and in 6,589 out of 49,264 (13.4%) of the DPP-4i (active control) group. The number of person-years-at-risk was 200,646 years and the longest duration of follow-up was 9.6 years.

**Figure 1:**
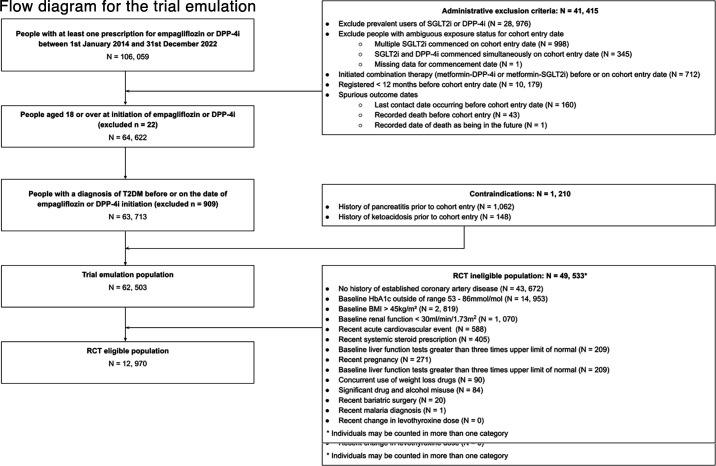
Flow diagram for the trial emulation. Flow diagram showing attrition of people according to eligibility criteria for the trial emulation. The trial emulation is a modified design of the EMPA-REG RCT and relaxes the stringent eligibility criteria of the RCT. BMI, body mass index; DPP-4i, dipeptidyl peptidase-4 inhibitors (alogliptin, linagliptin, sitagliptin, saxagliptin, vildagliptin); HbA1c, glycated hemoglobin A1c; RCT, randomized controlled trial; SGLT2i, sodium glucose co-transporter 2 inhibitor (empagliflozin, dapagliflozin, canagliflozin, ertugliflozin); T2DM, type 2 diabetes mellitus.

In the trial emulation population, 12,970 individuals (20.8%) would have met the eligibility criteria for the EMPA-REG RCT.^5^ A total of 13,239 patients with T2DM received a first prescription of empagliflozin within the study period, of whom 83.2% (n=11,011/13,239) would not have met the eligibility criteria for the EMPA-REG RCT. Most initiators of study drugs would have been excluded for not having established CVD at baseline (excluded n=43,672, 70.0%, figure 1) or having a recorded baseline HbA1c outside of the appropriate range (excluded n=14,953, 23.9%). There were 36,088 individuals (57.7% of trial emulation population) defined as RCT ineligible based on having a single exclusion criterion, with a further 13,445 people (21.5%) defined as RCT ineligible based on having more than one exclusion factor.

The trial emulation population was older (average age in trial emulation 64.0 years, standard deviation (SD) 13.5 vs average age in the RCT 63.1, SD 8.6, table 2), had substantially greater representation of women (42.5% of the population were female in the trial emulation vs 28% in the RCT, table 2), and had a higher baseline HbA1c compared with the RCT population (average baseline HbA1c in trial emulation: 74.3 mmol/mol, SD 17.8 vs 64.7 mmol/mol in the RCT, table 2).

**Table 2:** Demographics of the RCT and trial emulation populations, with further stratification according to RCT eligibility status in real-world population

		Trial emulation		
	EMPA-REG RCT (N=7020)	Overall population (N=62,503)	RCT eligible (N=12,970)	RCT ineligible (N=49,533)
Allocated to (RCT) or prescribed (trial emulation) empagliflozin (N, %)	4689 (66.8%)	13,239 (21.2%)	2228 (17.2%)	10,742 (21.7%)
Age (years)	63.1 (8.6)	64.0 (13.5)	70.8 (10.6)	62.2 (13.2)
Female (N, %)	1994 (28%)	26,535 (42.5%)	4561 (35.2%)	21,974 (44.4%)
Ethnicity (N, %)				
Missing White Asian Black Other Mixed	– 5089 (72.0%) 1518 (22.0%) 375 (5.0%) 70 (1.0%) –	35,739 (57.2%) 22,649 (36.2%) 2690 (4.3%) 819 (1.3%) 356 (0.6%) 250 (0.4%)	7752 (60.0%) 4673 (36.0%) 400 (3.1%) 76 (0.6%) 48 (0.4%) 21 (0.2%)	27,987 (56.5%) 17,976 (36.3%) 2290 (4.6%) 743 (1.5%) 308 (0.6%) 229 (0.5%)
Smoking status (N, %)				
Current Ex-smoker Non-smoker Missing	930 (13.0%) 3216 (46.0%) – –	9049 (14.5%) 28,409 (45.5%) 23,764 (38.0%) 1281 (2.0%)	1749 (13.5%) 7146 (55.1%) 3835 (30.0%) 240 (1.9%)	7300 (14.7%) 21,263 (42.9%) 19,929 (40.2%) 1041 (2.1%)
BMI (kg/m^2^)	30.6 (5.3)	32.5 (6.9)	31.0 (5.3)	32.9 (7.2)
HbA1c (mmol/mol)	64.7*	74.3 (17.8)	68.2 (8.6)	75.8 (19.1)
Systolic blood pressure (mm Hg)	135.0 (17.0)	133.5 (15.1)	133.2 (15.4)	133.6 (15.0)
Baseline estimated glomerular filtration rate (ml/min/1.73 m^2^)	74.0 (21.0)	82.9 (23.7)	74.4 (22.4)	85.23 (23.6)
Baseline low-density lipoprotein cholesterol (mg/dL)	2.2 (0.9)	2.5 (1.1)	2.3 (1.0)	2.6 (1.1)
Baseline high-density lipoprotein cholesterol (mg/dL)	1.2 (0.3)	1.2 (0.3)	1.13 (0.3)	1.16 (0.3)
Co-prescribed medication (N, %)				
Metformin Sulfonylurea Insulin GLP-1 receptor agonists Statin/lipid-lowering agent Anti-hypertensive agent	5193 (74.0%) 3006 (42.8%) 3387 (48.2%) 196 (2.8%) 5387 (77%) 6641 (94%)	41,397 (66.2%) 20,966 (33.5%) 5453 (8.7%) 1968 (3.1%) 41,863 (67.0%) 37,300 (59.7%)	9006 (69.4%) 5004 (38.6%) 1396 (10.8%) 389 (3.0%) 10,849 (83.6%) 10,170 (78.4%)	32,391 (65.4%) 15,962 (32.2%) 4057 (8.2%) 1579 (2.5%) 31,014 (62.6%) 27,130 (54.8%)

Table comparing the randomized controlled trial (RCT) and the trial emulation population, with further stratification by RCT eligibility status. Data presented as mean (SD) or count (column percentage) for consistency with trial reporting.

* SD not provided for HbA1c mmol/mol unit.

BMI, body mass index; DPP-4i, dipeptidyl peptidase-4 inhibitors (alogliptin, linagliptin, sitagliptin, saxagliptin and vildagliptin); eGFR, estimated glomerular filtration rate; HbA1c, glycated haemoglobin A1c; RCT, randomized controlled trial; SGLT2, sodium glucose co-transporter 2 inhibitor (empagliflozin, dapagliflozin, canagliflozin and ertugliflozin); T2DM, type 2 diabetes mellitus.

The trial emulation cohort had a lower burden of co-prescribed medication compared with the RCT population (table 2). For example, much greater numbers were co-prescribed insulin in the RCT compared with the trial emulation (3,387 people in the RCT, 48.2% of RCT cohort vs 54,53 people in the trial emulation, 8.7%). Similar patterns can be seen across most anti-diabetes drug classes and anti-hypertensive agents. This likely reflects different burdens of diseases between RCT and real-world populations, but also geographical variations in clinical practice.

As expected in an RCT, the empagliflozin and control groups are approximately balanced for all key characteristics. However, in the trial emulation population, people who commenced empagliflozin tended to be younger but have worse metabolic markers (higher average BMI and higher average HbA1c) compared with people initiated on DPP-4i (online supplemental material 8). People in the empagliflozin group tended to have lower burden of comorbidities—particularly for CVD (present in 3424/13,239, 25.7% of the empagliflozin group vs 15,407/49,264, 31.3% of the DPP-4i group) and dementia (present in 50/13,239, 0.4% of the empagliflozin group vs 1172, 2.4% of the DPP-4i group). In addition, people in the empagliflozin group were less likely to be prescribed metformin, sulfonylurea, anti-hypertensives, and lipid-lowering drugs (online supplemental material 8).

Real-world users of empagliflozin, who would be RCT ineligible, tended to be younger, more likely to be female, have less co-prescribed medication and fewer comorbidities, but worse metabolic health (higher HbA1c, higher BMI) compared with real-world users of empagliflozin who are RCT eligible. This suggests that real-world populations have less burden of comorbidities and are generally healthier than those who are represented in the RCT, although with worse metabolic status (online supplemental material 8).

### Trial emulation outcome analysis

**Figure 2:**
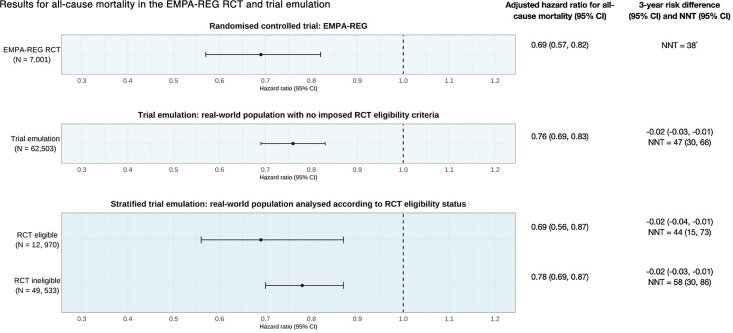
Real-world effects of empagliflozin on all-cause mortality*.* Forest plot showing the HR and 95% CIs for all-cause mortality, 3-year risk difference, and number needed to treat (NNT) to prevent one death in the randomized controlled trial (RCT), trial emulation, and stratified trial emulation analysis. The trial emulation population represents initiators of study drugs (empagliflozin or DPP-4i), without imposing RCT-defined eligibility criteria. The stratified trial emulation refers to an adjusted Cox proportional hazards model where there is an additional interaction between RCT eligibility status and empagliflozin, allowing estimation of the HR for empagliflozin versus DPP-4i in both RCT eligible and RCT ineligible real-world populations. *Risk difference and 95% CI not published in post hoc analysis of the EMPA-REG RCT*.* DPP-4i, dipeptidyl peptidase-4 inhibitors.

In the trial emulation, the primary outcome of all-cause mortality occurred in 551 out of 13,239 (4.2%) of the empagliflozin group and in 6,589 out of 49,264 (13.4%) of the active control group. The adjusted HR for all-cause mortality in the Cox proportional hazards model was 0.76 (95% CI 0.69 to 0.83), providing strong evidence of a mortality benefit for people initiated on empagliflozin compared with those who initiated DPP-4i (figure 2).

There was a reduced 3-year risk of mortality in the empagliflozin group compared with the DPP-4i group (risk difference −0.02, 95% CI −0.03 to –0.01, figure 2). The corresponding NNT is 47 people (95% CI 30 to 66, figure 2). The IPTW analysis yielded similar results (online supplemental material 11) for both the estimate as an average treatment effect and average treatment effect in the treated.

### Trial emulation sensitivity analysis

The E-value for treatment assignment was risk ratio 1.98 (95% CI 1.70 to 2.27, online supplemental material 10). This suggests that an unmeasured confounder would need to be strongly associated with both treatment and outcome (risk ratio ≥1.98) to fully explain the observed association. It is unlikely that a potential confounder with such a strong association with both treatment and the outcome would have been overlooked in the study. This supports the robustness of the treatment–outcome association against unmeasured confounding.

### Stratified trial emulation to assess treatment effect by RCT eligibility

HRs for all-cause mortality were consistent between RCT-eligible and RCT-ineligible initiators of empagliflozin (figure 1), with no evidence of an interaction between the treatment effect of empagliflozin and RCT eligibility status (p-value for interaction 0.27). The RCT-eligible strata-specific hazard ratios met all predefined agreement criteria with the published RCT results, confirming the successful benchmarking of the observational findings against the RCT findings.

The NNT at 3 years from the RCT was within the 95% CIs for all trial emulation estimates, including both RCT-eligible and RCT-ineligible populations (figure 2). There was no significant difference in estimates of the HR for all-cause mortality and corresponding NNT between the RCT eligible and ineligible population (figure 2).

## Discussion

In this study, we applied a trial emulation framework to investigate the real-world treatment effect of empagliflozin in people with T2DM. Our findings confirm that the mortality benefits of empagliflozin observed in the EMPA-REG RCT are realized in real-world practice. Notably, we found that the majority of real-world empagliflozin users would not have met the eligibility criteria imposed by the EMPA-REG RCT. By modifying the design of the EMPA-REG RCT to reflect contemporary real-world utilization, we demonstrate that individuals excluded from the RCT, but now receiving treatment with empagliflozin, have comparable treatment effect to those who were represented in the RCT. This provides a novel evidence base for people with T2DM, many of whom are under-represented and ultimately underserved by RCTs.

It is well recognized that RCTs often have limited external generalizability, often due to strict eligibility criteria.^29^ A key finding of the present study is that as few as 16.8% of individuals with T2DM in the UK initiated on empagliflozin would have met the original EMPA-REG RCT eligibility criteria. This aligns with findings from a Taiwanese study, where only 18.7% of SGLT2i users met relevant RCT eligibility criteria.^30^ Differences between RCT populations and treated patients reflect the wider treatment scope recommended by clinical guidelines like those from NICE. These guidelines now recommend SGLT2i use in people with lower burden of CVD, or those with risk factors for CVD.^19^

Our real-world evidence demonstrates consistent mortality benefits across both RCT eligible and ineligible real-world populations, many of whom have low burden, or absence of, CVD. The CIs for the all-cause mortality HR and NNT overlap, suggesting that there was no strong evidence of a significant difference in treatment effects in populations according to their RCT eligibility status.

This is a clinically important finding as it addresses a gap in the current evidence-base for empagliflozin, identified by both NICE committee deliberations and others.^2 9 31^ A previous meta-analysis of 7 RCTs (n=4, 495) studying the effects of empagliflozin versus placebo in people with T2DM with low-medium burden of CVD concluded that there was no evidence supporting significant reduction in all-cause mortality (HR 0.67, 95% CI 0.28 to 1.63).^32^ However, in the present study we show a significant and long-term mortality benefit for empagliflozin in a diverse and clinically relevant population of people with T2DM. The difference between these findings and the present study likely reflects the larger population studied in the real-world analysis, providing greater precision.

### Comparison with other studies

Two prior trial emulations replicated the EMPA-REG RCT using insurance-based observational data, but did not extend to the wider population considered in the present study. A US-based study using an as-treated approach with 1:1 propensity score matching on over 1000 covariates successfully replicated the RCT HR for 3P-MACE.^27^ Similarly, a South Korean study used an intention-to-treat approach comparing empagliflozin to sitagliptin and reached all predefined agreement metrics.^33^ We advance existing trial emulations by studying the robustness of real-world effects to modifications in trial eligibility criteria, which addresses key gaps in the current T2DM evidence base. This facilitates a better understanding of longer-term, real-world treatment effects in representative populations and supports evidence generation that is directly applicable to real-world clinical decision-making.

### Strengths and limitations of this study

This study uses a large, representative UK primary care database, benefiting from comprehensive health record capture.^13 14^ Given that most diabetes care occurs in primary care settings with incentives for recording key covariates, data completeness is high. The study period (2014–2022) coincided with variable prescribing practices due to a lack of clear oral anti-diabetic guidelines, helping to reduce systematic bias between groups.^23^

A key aspect of the study design was the emulation of the EMPA-REG RCT in UK primary care data. To our knowledge, this is the first such emulation in a European setting. We were able to successfully benchmark the results of our real-world analysis to the published results of the RCT, giving us confidence that our analysis is appropriately handling confounding. In addition, this design helps avoid issues in other similar observational studies of T2DM medication such as immortal time bias and time-lag bias.^31^ We acknowledge the potential for residual confounding, for example, by unmeasured covariates such as frailty. However, we do not believe that the presence of unmeasured confounding would be so substantial as to alter the conclusions of this study because of our rigorous modeling strategy, close replication of the existing RCT findings, and supportive quantitative bias assessment (E-value analysis). The EMPA-REG RCT was a placebo-controlled trial, and to approximate this in observational settings, we compared new-user initiators of empagliflozin with an active comparator, new-user initiators of DPP-4i. We believe this helps to reduce confounding by indication and is the most approximate comparator to proxy the EMPA-REG RCT. We also acknowledge that the primary outcome of the trial emulation is a secondary outcome of the EMPA-REG RCT, but cause-specific mortality data were not available within the electronic health record database. This study generated real-world evidence within a UK context, and while likely generalizable to other developed countries, confirmation in more diverse settings would be valuable.

This trial emulation did not assess safety outcomes, an important area for future research. Real-world safety signals may differ from RCT findings due to greater comorbidities and polypharmacy, increasing the risk of drug interactions and adverse effects.^34^

## Conclusions

The mortality benefit of empagliflozin, originally seen in the EMPA-REG RCT, is also observed in real-world settings, among a broader, more clinically relevant population of people with T2DM. Although most individuals prescribed empagliflozin in routine care would not have qualified for the RCT, our findings demonstrate a consistent mortality benefit across both RCT-eligible and RCT-ineligible people. This study provides robust real-world evidence to support the wider utilization of empagliflozin in T2DM management, beyond the narrow eligibility criteria imposed by RCTs. It also highlights the value of trial emulation and real-world data as complementary approaches to RCTs, offering novel insights where RCT evidence is limited or lacking.

## Supplementary material

10.1136/bmjdrc-2025-005672 online supplemental file 1

## Data Availability

Data may be obtained from a third party and are not publicly available.
